# Editorial: Therapeutic approaches in venous thromboembolism management and coagulation

**DOI:** 10.3389/fmed.2025.1719487

**Published:** 2025-10-24

**Authors:** Carmine Siniscalchi, Pierpaolo Di Micco

**Affiliations:** ^1^Internal Medicine Ward, University of Parma, Parma, Italy; ^2^Internal Medicine Ward, “P.O. Santa Maria delle Grazie”, ASL Naples 2, Pozzuoli, Naples, Italy

**Keywords:** venous thromboembolism, therapies, editorial, coagulation, management

This Research Topic focuses on the daily management of venous thromboembolism, from prophylaxis to treatment and prognosis.

Several scholars reported their clinical experiences in different clinical settings where constant updates are necessary.

Alsuhebany et al. reported an intriguing experience with different types of thromboprophylaxis for inpatients in the neurological intensive care unit with hematological/oncological malignancies. It is difficult to determine the best practice for patients at risk of developing thrombosis due to bedridden status and malignancies associated with frequent thrombocytopenia or platelet dysfunction from oncological treatments, and this topic is still under discussion (Alsuhebany et al.) (1). Often, this clinical scenario can benefit from nursing surveillance because of the instability of clinical conditions of patients with VTE or at risk for VTE. As in other aspects of the daily clinical management of patients, from acute treatments for VTE to prolonged anticoagulation, the nursing system is acquiring a fundamental role (Diao et al.). In oncological management, in fact, nursing support is essential. Particular interest, in fact, is reserved for cancers at high risk of VTE, such as different types of lung cancer, as reported by Xie et al., Chen et al., and Liu et al. Surveillance is high for both patients who have undergone surgery and those undergoing chemotherapy. The clinical characteristics of patients with lung cancer who develop VTE may also differ based on oncological characteristics and the presence of other thrombotic risk factors (Xu et al.). Specific characteristics of patients with VTE represent a shift from evidence-based medicine to tailored medicine, and among the relevant aspects that may influence the prognosis of patients with VTE, PaCo2 levels and the location of pulmonary thrombus may identify a subgroup of patients with a poor prognosis (Demsie et al.); moreover, inherited predisposition appears to play a specific role also in these vulnerable patients as evidenced by data found during studies on the *TMEM132A* gene (Xie et al.).

However, patients with major trauma who undergo major orthopedic surgery may also receive tailored treatments: prophylaxis of post-traumatic hemorrhage with tranexamic acid administered before hospitalization (Li et al.) represents a constant effort to decrease mortality in this critical clinical setting, although it could increase the risk of future VTE in major orthopedic surgery. This clinical setting is, in fact, historically associated with an increased risk of thrombotic complications, including hip and knee prosthesis, as reported by Ren et al.

However, the optimal anticoagulation during hospitalization and in the weeks following an acute VTE is still a matter of clinical debate, due to the constant risk of prolonged management of anticoagulant treatment (2) (Yang and Liu; Huang et al.; Siniscalchi et al.).
